# Clinical course and role of embolization in patients with spontaneous rupture of hepatocellular carcinoma

**DOI:** 10.3389/fonc.2022.999557

**Published:** 2022-09-05

**Authors:** Juil Park, Yun Soo Jeong, Yun Seok Suh, Hyo-Cheol Kim, Jin Wook Chung, Jin Woo Choi

**Affiliations:** ^1^ Department of Radiology, Severance Hospital, Seoul, South Korea; ^2^ Department of Radiology, Seoul National University Hospital, Seoul, South Korea; ^3^ Department of Radiology, Seoul National University College of Medicine, Seoul, South Korea

**Keywords:** hepatocellular carcinoma, spontaneous rupture, transcatheter arterial embolization, transcatheter arterial chemoembolization, intraperitoneal drop metastasis

## Abstract

**Background:**

A diverse clinical course after the spontaneous rupture of hepatocellular carcinoma (HCC) renders nonstandardized treatment protocols.

**Purpose:**

To evaluate clinical course and role of transcatheter arterial embolization (TAE) in patients with rupture of HCC.

**Materials and methods:**

This retrospective study included 127 patients who were treated for ruptured HCC at single institution between 2005 and 2014. After multidisciplinary discussion, patients underwent medical management, TAE, emergency surgery or staged surgery. Patients were retrospectively divided into two groups based on the intent of treatment: curative and palliative. The rebleeding rate and 1-month and overall survival (OS) were compared between two groups. The incidence and survival of patients with intraperitoneal drop metastasis (IPDM) were also analyzed.

**Results:**

The overall rebleeding rate in patients who underwent TAE was 3.1% (3/96). One-month mortality rate was 6.3% (8/127). The rebleeding and 1-month mortality rates were not significantly different between two groups. OS was significantly higher in the curative treatment group (median: 12.0 vs 2.2 months, *p*<0.001). Among 96 patients who initially received TAE, ten patients underwent staged operation (10.4%). The median OS for medical management, TAE, emergency surgery and staged surgery was 2.8, 8.7, 19.1 and 71.1 months, respectively. Of all patients, 15.2% developed IPDM mostly within 1 year and their survival was poorer than that of patients without IPDM (median: 6.3 vs. 15.1 months, *p*<0.001).

**Conclusion:**

TAE provided effective immediate hemostasis with a low rebleeding rate and may serve as a bridge to elective surgery. IPDM frequently occurred within 1 year and manifested poor survival; thus, close surveillance should be considered for patients with spontaneous rupture of HCC.

## Introduction

Spontaneous rupture of hepatocellular carcinoma (HCC) can manifest a wide spectrum of symptoms, from mild abdominal pain to abrupt hypovolemic shock, which result in a diverse clinical course. Its reported mortality rate is 25-75% (1, 2). Owing to the hypervascularity of HCC, timely intervention, including hemostasis and volume resuscitation in the acute phase, is vital to achieve hemodynamic stability in patients with ruptured HCC (3). Spontaneous rupture of the tumor also affects patients’ long-term management plans even after successful recovery from acute hemodynamic instability. The incidence of intraperitoneal drop metastasis (IPDM) increases after HCC rupture because of potential tumor cell spillage in the peritoneal space, which hinders curative treatments after acute management (4–6). Therefore, patient management should be individualized in consideration of the initial manifestation, hepatic functional reserve, operability, and chance of IPDM.

Some studies have suggested treatment algorithms, but because of the lack of high-level evidence, the best treatment approach is still controversial, and patients with ruptured HCCs are largely treated using local protocols (3). Considering the high mortality rate (85-100%), conservative management should be reserved for patients in whom transarterial embolization (TAE) and surgery are not feasible because of poor liver function and advanced tumor stage (7, 8). Emergency surgery and TAE are the two main treatment modalities used to achieve immediate hemostasis. While surgery is advantageous for both hemostasis and tumor resection in a single operation procedure, TAE is less invasive, shows a relatively high success rate of hemostasis in the acute phase (53–100%) and has a lower 30-day mortality rate than surgery (0–37% vs. 28–75%) (9–13).

Considering the diverse clinical course following HCC rupture and various roles of TAE in each case, the effectiveness of TAE should be scrutinized separately for each situation. In addition, as the probability of IPDM is relatively high after spontaneous rupture of HCC, a longitudinal study on the development of IPDM is warranted. Therefore, this retrospective study was conducted to determine the role of TAE in acute management of HCC rupture and evaluate the development and clinical impact of IPDM.

## Materials and methods

### Patients

**Figure 1:**
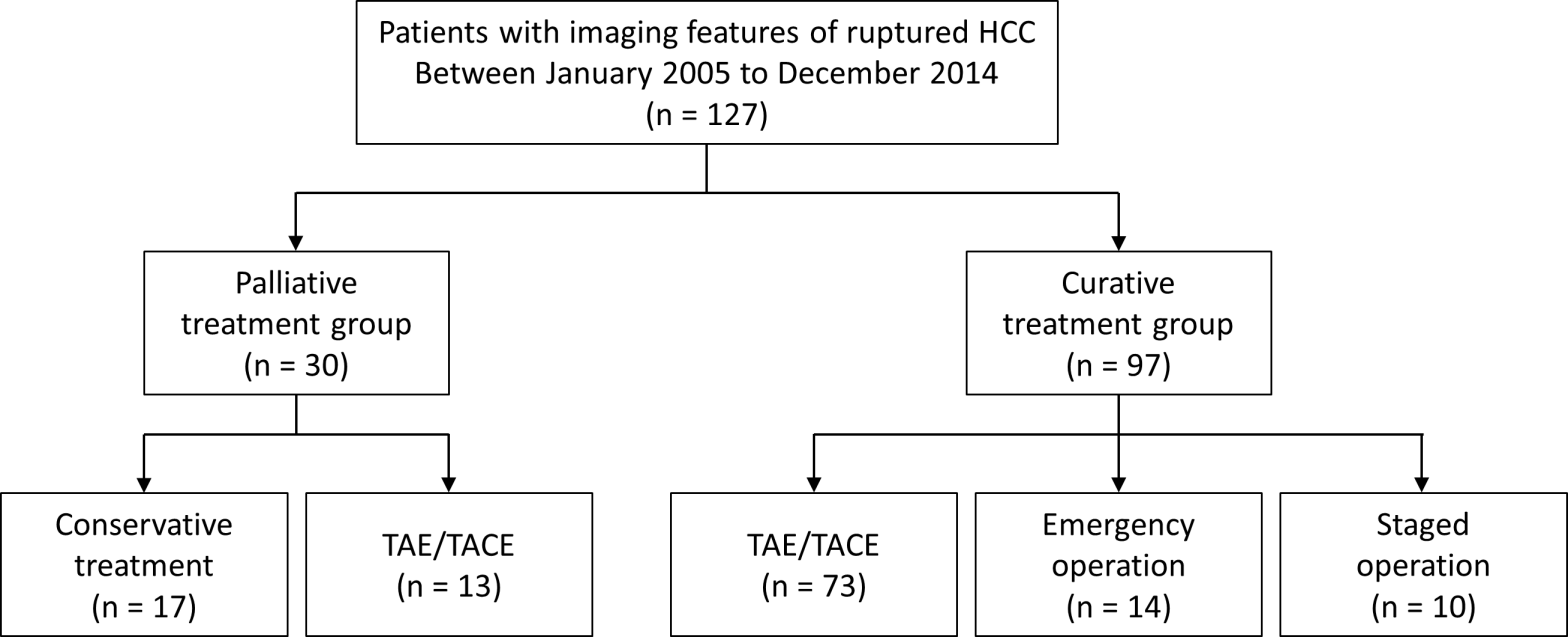
Flow chart of the study population. HCC, hepatocellular carcinoma; TAE, transcatheter arterial embolization; TACEm transcatheter arterial chemoembolization.

This retrospective study was approved by the institutional review board and the requirement for informed consent was waived. All patients diagnosed with HCC were retrospectively recorded in the institutional cohort, and the electronic database contained imaging findings from initial and subsequent studies. Among the 10536 patients who were initially diagnosed with HCC between January 2005 and December 2014, 133 were recorded as having imaging features suggestive of ruptured HCC. The patients’ images were reviewed, and the records of 127 patients who met the imaging criteria for a ruptured HCC were finally analyzed (**Figure 1**).

### Imaging evaluation of ruptured HCC

All patients underwent multiphase dynamic computed tomography (CT) or magnetic resonance (MR) imaging. The images were reviewed by two interventional radiologists (H.C.Kim and J.W.Choi with 15 and 4 years of experience in interventional oncology) in consensus. Ruptured HCC was diagnosed when a liver tumor with arterial-phase enhancement and portal- or delayed-phase washout abutted the liver surface and had one or more of the following findings: contrast media extravasation from the tumor to the peritoneum, tumor protrusion with hemoperitoneum, and focal discontinuity of the tumor surface with hemoperitoneum (14). In patients with ruptured HCC, the largest tumor size, number, distribution (unilobar and bilobar), vascular invasion, imaging signs of portal hypertension (ascites, varix, splenomegaly > 12 cm), extrahepatic spread of HCC, and IPDM were also evaluated. IPDM was defined as a hypervascular intraperitoneal mass with or without central necrosis and engorgement of adjacent omental vessels (6). Two interventional radiologists also reviewed all follow-up liver CT and MRI scans of each patient to identify the occurrence of new or additional IPDMs.

### Treatment of ruptured HCC

Tumor bleeding was initially managed by volume resuscitation, transfusion, or inotropic agents at the discretion of the attending physician. Emergency embolization was performed in patients with hemodynamic instability. Patients with terminal stage HCC and stable vital signs received supportive care only. Surgical resection was considered as the initial treatment for operable patients with stable vital signs. Operability was determined by consultation with hepatic surgeons or a multidisciplinary team. Interventional management was administered to patients who were not eligible for either surgery or supportive care. For these patients, interventional radiologists considered the vital signs, cancer stages, and liver functions, and performed transarterial chemoembolization (TACE) or TAE. TACE using iodized oil (Lipiodol Ultra-Fluid; Guerbet, Aulnay‐Sous‐Bois, France) plus doxorubicin chemoemulsion was primarily considered for most candidates, while TAE was preferred for patients with Child-Pugh class B or C, or hemodynamic instability. For both TACE and TAE, gelatin sponge particles were primarily used as embolic materials, but cyanoacrylate and iodized oil mixtures were also considered when contrast extravasation was evident on digital subtraction angiography and a microcatheter was advanced into the culprit arteries. After initial TACE or TAE, the operability of the patients was reassessed by a multidisciplinary conference of surgeons and interventional radiologists, and operable patients were treated with hepatic resection. The remaining patients were managed with subsequent TACE, systemic therapy, or supportive care at the discretion of the hepatologists.

### Clinical data evaluation

Based on the review of electronic medical records, the management goal of ruptured HCCs was divided as curative treatment group and palliative treatment group. Surgical resection, staged surgical resection following TACE, and most TACE procedures were regarded as curative treatment, while selective TACE or TAE only for bleeding foci, and supportive care were considered as palliative treatment. With regard to the clinical course, two interventional radiologists (J.W.Chung and H.C.Kim with 27 and 15 years of experience in interventional oncology, respectively) reviewed all accessible medical records and images after tumor rupture to identify radiologically diagnosed rebleeding within 1 month, new development of IPDM within 5 years, and overall survival (OS). Anonymized survival data from the rupture of HCC were acquired from the Ministry of Interior and Safety of South Korea, which archives all citizens’ survival data and updates them daily.

### Statistical analysis

Demographic data of the curative treatment group and palliative treatment group were compared using the independent t-test and chi-square test. The 1-month rebleeding rates after interventional management with curative treatment (TACE) and palliative treatment (selective TACE only for bleeding foci, TAE) were compared using Fisher’s exact test. The occurrence of IPDM in all patients and OS in each treatment group were evaluated using the Kaplan-Meier method and log-rank test. In the curative treatment group, the OS depending on each modality (TAE/TACE, surgery, and staged surgery) was also estimated for all patients. Statistical significance was set at *p* < 0.05. All statistical analyses were performed using SPSS statistical software version 25.0 (IBM Corp., Armonk, NY, USA).

## Results

### Baseline patient characteristics

Among 127 patients with ruptured HCC, 97 and 30 were managed by curative treatment and palliative treatment, respectively (**Table 1**). Baseline laboratory findings, such as albumin, bilirubin, aspartate aminotransferase, and alkaline phosphatase levels were worse in the palliative treatment group (all *p* < 0.05). A higher proportion of patients in the palliative treatment group had ascites, portal hypertension and Child Pugh classes B and C (all *p* < 0.001). In terms of tumor characteristics, greater size, more infiltrative type of tumor, and more bilobar distribution were noted in the palliative treatment group (all *p* < 0.01). All these differences noted between the two groups were expected, considering that the patients in the palliative treatment group were treated with hemostasis as the main objective. Poor Child Pugh class, presence of ascites, portal hypertension, bilobar tumor distribution, and more infiltrative tumor type were noted in patients who underwent interventional treatment rather than emergency or staged surgery (all *p* < 0.05) (**Table 2**). Twelve patients (14.0%) who underwent TAE/TACE demonstrated contrast extravasation on digital subtraction angiography. In these cases, cyanoacrylate and iodized oil mixture was applied selectively to control the active bleeding.

**Table 1:** Characteristics of the study sample between curative and palliative treatment groups.

	Curative treatment group (n = 97)	Palliative treatment group (n = 30)	P value
**Age**	58 ± 13	59 ± 11	0.989
**Sex (M/F)**	88 (90.7)/9 (9.3)	26 (86.7)/4 (13.3)	0.504
**Etiology** ** HBV** ** HCV** ** Alcohol**	60 (61.9) 7 (7.2) 18 (18.6)	26 (86.7) 2 (6.7) 1 (3.3)	**0.013** 1.000 **0.043**
**Laboratory value**			
** Platelet (×10^3^/μℓ)**	223 ± 103	210 ± 98	0.531
** Albumin (g/dL)**	3.6 ± 0.6	3.0 ± 0.7	**<0.001**
** Total bilirubin (mg/dL)**	1.3 ± 0.8	2.2 ± 1.8	**0.011**
** PT (INR)**	1.15 ± 0.18	1.33 ± 0.49	0.060
** Creatinine (mg/dL)**	1.12 ± 0.73	1.13 ± 0.53	0.956
** AST (IU/L)**	112 ± 191	211 ± 232	**0.021**
** ALT (IU/L)**	70 ± 77	90 ± 89	0.231
** ALP (IU/L)**	133 ± 112	185 ± 123	**0.029**
** GGT (IU/L)**	201 ± 282	303 ± 177	0.174
** AFP (ng/mL)**	39016 ± 127314	97368 ± 339723	0.182
** PIVKA (mAU/mL)**	21634 ± 41490	39103 ± 34867	0.245
** Ascites (absent/present)**	13 (13.4) /84 (86.6)	16 (53.3)/14 (46.7)	**<0.001**
**Portal hypertension (absent/present)**	32 (33.0) /65 (67.0)	22 (73.3)/8 (26.7)	**<0.001**
**Child Pugh class** ** A** ** B** ** C**	77 (79.4) 19 (19.6) 1 (1.0)	9 (30.0) 15 (50.0) 6 (20.0)	**<0.001**
**Tumor size**	9.4 ± 4.1	12.0 ± 5.5	**0.006**
**Tumor type** ** Single nodular** ** Multinodular** ** Infiltrative**	47 (48.5) 20 (20.6) 30 (30.9)	6 (20.0) 5 (16.7) 19 (63.3)	**0.004**
**Tumor distribution (uni-/bilobar)**	66 (68.0)/31 (32.0)	7 (23.3)/23 (76.7)	**<0.001**

HBV, hepatitis B virus; HCV, hepatitis C virus; PT, prothrombin time; AST, aspartate aminotransferase; ALT, alanine aminotransferase; ALP, alkaline phosphatase; GGT, gamma glutamyl peptidase; AFP, alpha fetoprotein; PIVKA, prothrombin-induced by vitamin K absence or antagonist; AU, arbitrary unit.

The bold values are the parameters of statistically significant differences between the two groups.

**Table 2:** Characteristics of the study sample undergoing transcatheter arterial embolization or surgery in the curative treatment group.

	TAE/TACE (n = 73)	Emergency and Staged Surgery (n = 24)	P value
**Age**	59 ± 13	56 ± 13	0.317
**Sex (M/F)**	66 (90.4)/7 (9.6)	22 (91.7)/2 (8.3)	1.000
**Etiology** ** HBV** ** HCV** ** Alcohol**	47 (64.4) 5 (6.8) 16 (21.8)	13 (54.2) 2 (8.3) 2 (8.3)	0.239 1.000 0.225
**Laboratory value**			
** Platelet (×10^3^/μℓ)**	221 ± 109	228 ± 86	0.597
** Albumin (g/dL)**	3.5 ± 0.6	3.9 ± 0.5	**0.008**
** Total bilirubin (mg/dL)**	1.3 ± 0.7	1.2 ± 0.9	0.363
** PT (INR)**	1.16 ± 0.20	1.10 ± 0.09	0.130
** Creatinine (mg/dL)**	1.17 ± 0.82	0.98 ± 0.25	0.297
** AST (IU/L)**	126 ± 216	72 ± 57	**0.047**
** ALT (IU/L)**	73 ± 82	59 ± 57	0.431
** ALP (IU/L)**	143 ± 121	102 ± 67	**0.043**
** GGT (IU/L)**	236 ± 318	103 ± 93	0.118
** AFP (ng/mL)**	37574 ± 117588	43537 ± 157050	0.850
** PIVKA (mAU/mL)**	16744 ± 24966	32848 ± 65751	0.407
**Ascites (absent/present)**	60 (85.2)/13 (17.8)	24 (100.0)/0 (0.0)	**0.034**
**Portal hypertension (absent/present)**	42 (57.5)/31 (42.5)	23 (95.8)/1(4.2)	**<0.001**
**Child Pugh class** ** A** ** B** ** C**	52 (71.2) 20 (27.4) 1 (1.4)	23 (95.8) 1 (4.2) 0 (0.0)	**0.044**
**Tumor size**	9.7 ± 4.3	8.4 ± 3.4	0.144
**Tumor type** ** Single nodular** ** Multinodular** ** Infiltrative**	29 (39.8) 15 (20.5) 29 (39.8)	16 (66.7) 5 (20.8) 3 (12.5)	**0.032**
**Tumor distribution (uni-/bilobar)**	44 (60.2)/29 (39.8)	20 (83.3)/4 (16.7)	**0.048**

TAE, transarterial embolization; TACE, transcatheter arterial chemoembolization; HBV, hepatitis B virus; HCV, hepatitis C virus; PT, prothrombin time; AST, aspartate aminotransferase; ALT, alanine aminotransferase; ALP, alkaline phosphatase; GGT, gamma glutamyl peptidase; AFP, alpha fetoprotein; PIVKA, prothrombin-induced by vitamin K absence or antagonist; AU, arbitrary unit.

The bold values are the parameters of statistically significant differences between the two groups.

In the curative treatment group, 14 (14.4%, 14/97), 10 (10.3%, 10/97), and 73 (75.3%, 73/97) patients underwent surgical resection as initial treatment, staged surgical resection following TACE, and TACE, respectively. In the palliative treatment group, 13 (43.3%, 13/30), and 17 (56.7%, 17/30) patients received TACE/TAE and supportive care, respectively (**Supplementary Table 1**).

### Short-term outcome: Rebleeding and death within 1-month

Early rebleeding within 1-month from the initial management was observed in three patients (2.4%, 3/127); of these, two patients (2.1%, 2/97) were from the curative treatment group and one patient (3.3%, 1/30) was from the palliative treatment group (**Supplementary Table 1**). A total of 96 patients were initially treated with TACE or TAE, and the 1-month rebleeding rate of interventional management was 3.1% (3/96). Specifically, two (2.4%, 2/83) patients in the curative treatment group and one (7.7%, 1/13) in the palliative treatment group experienced rebleeding within 1 month, but the rebleeding rates were not significantly different (*p* = .357).

Eight out of 127 patients (1-month mortality rate, 6.3%) died 4–28 days after HCC rupture (median, 14.5 days): three (3.1%, 3/97) patients from the curative treatment group and five (16.7%, 5/30) from the palliative treatment group (*p* = 0.018) (**Supplementary Table 1**). Among the five patients (5.2%, 5/96) who underwent TACE or TAE for bleeding control, three patients died of early rebleeding after initial hemostasis. The remaining two patients succumbed to multiorgan failure, including severe deterioration of liver function.

### Long-term outcome: Overall survival and IPDM

**Figure 2:**
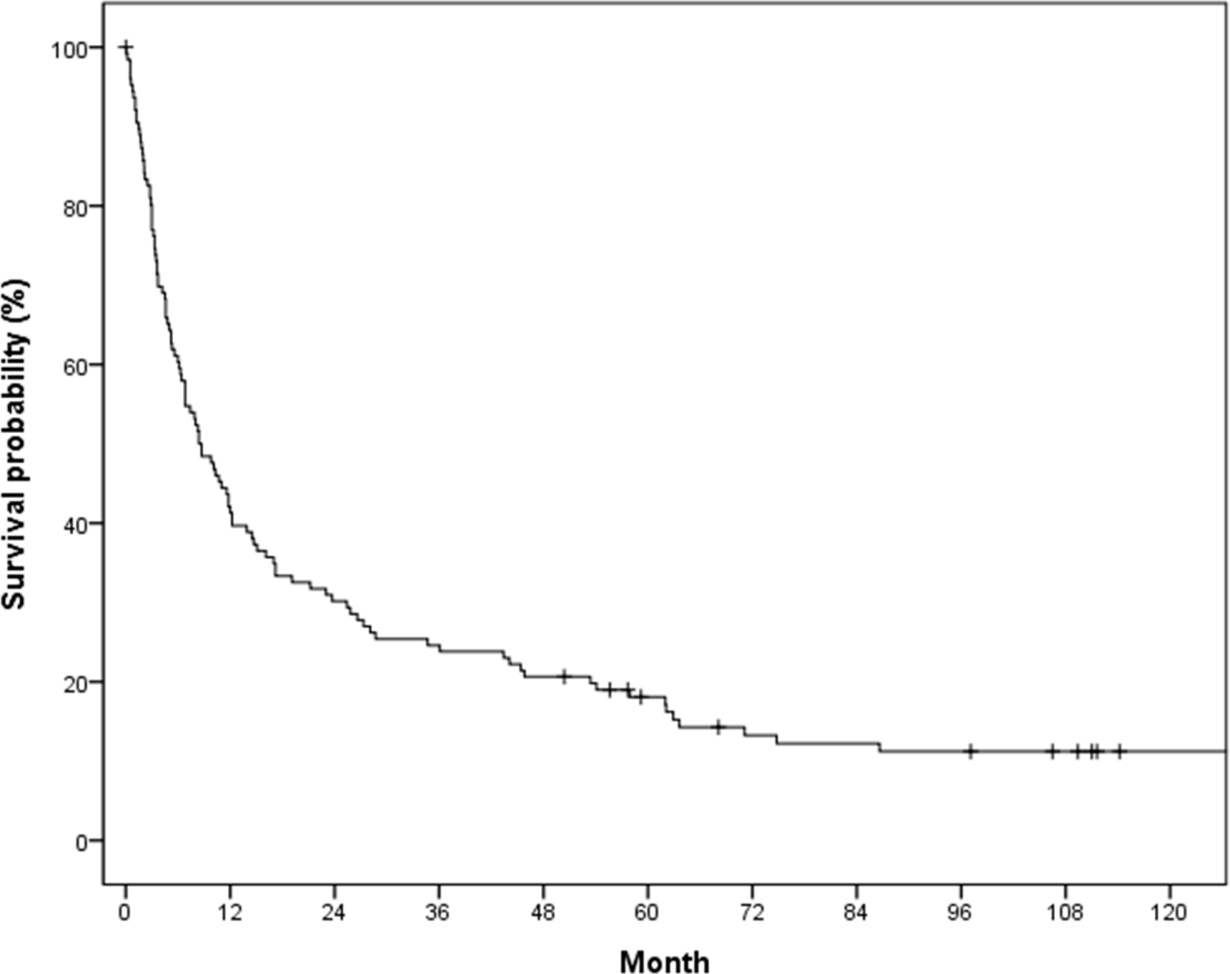
Overall survival in the study population.

**Figure 3:**
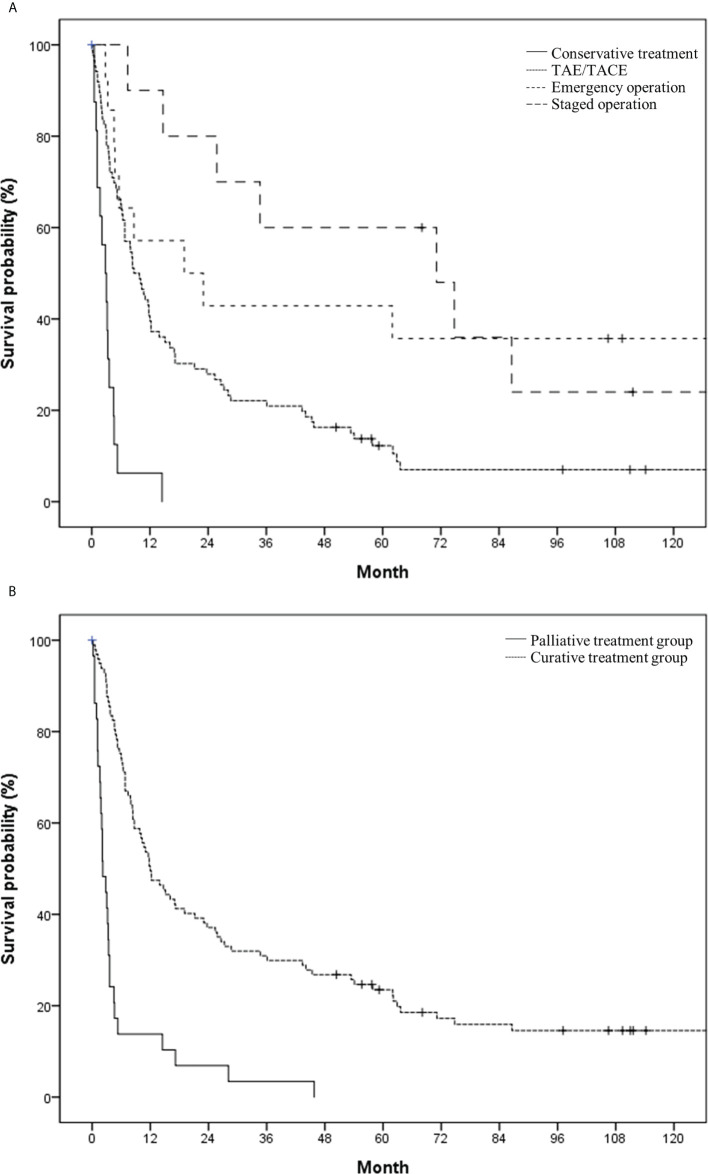
Overall survival in the study population based on **(A)** initial treatment modality (conservative treatment vs. transcatheter arterial (chemo-) embolization vs. emergency operation vs. staged operation) and **(B)** treatment intent (palliative vs. curative).

The median survival time and 1-year survival rate following HCC rupture were 8.4 months and 41.3%, respectively (**Figure 2**). The median survival of patients with conservative treatment only, TAE/TACE, emergency operation, and staged hepatectomy was 2.8, 8.7, 19.1 and 71.1 months, respectively (**Figure 3A**). Except for the comparison between patients who underwent emergency surgery and staged hepatectomy (*p* = 0.606), these differences were statistically significant (all *p* < 0.05). OS was significantly longer in the curative treatment group (median, 12.0 months) than in the palliative treatment group (median, 2.2 months) (p <.001) (**Figure 3B**).

**Figure 4:**
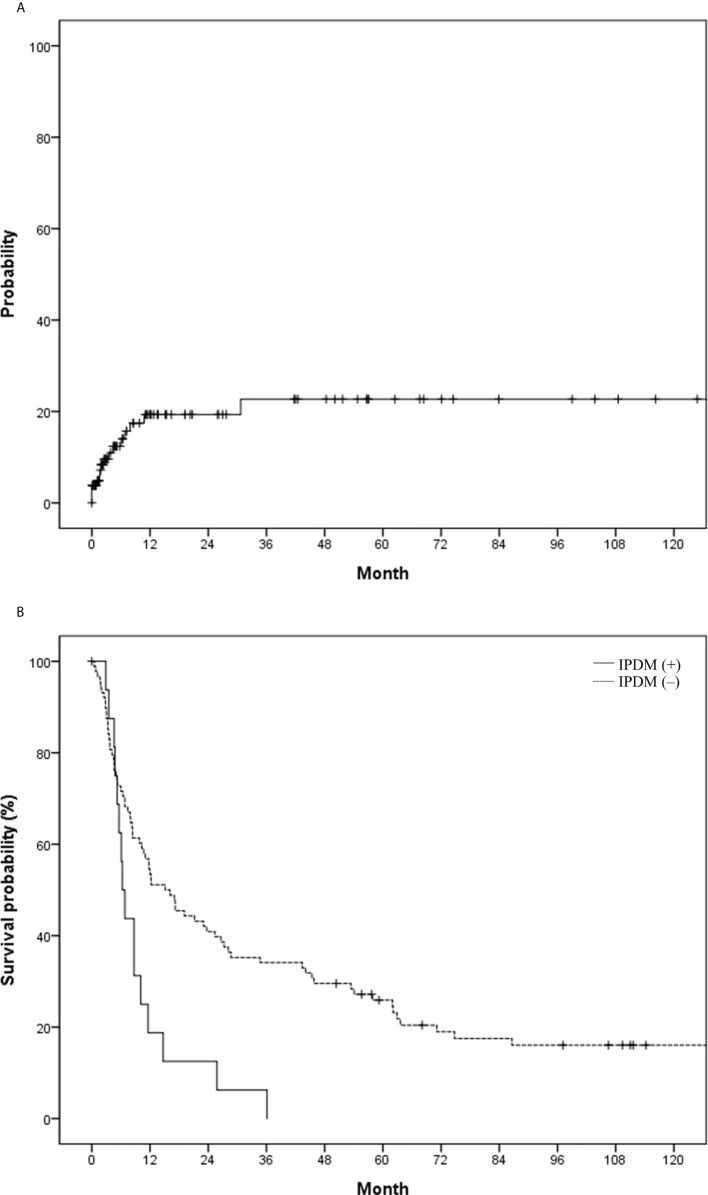
**(A)** Probability of intraperitoneal drop metastasis (IPDM) and **(B)** overall survival in the study population based on IPDM.

**Figure 5:**
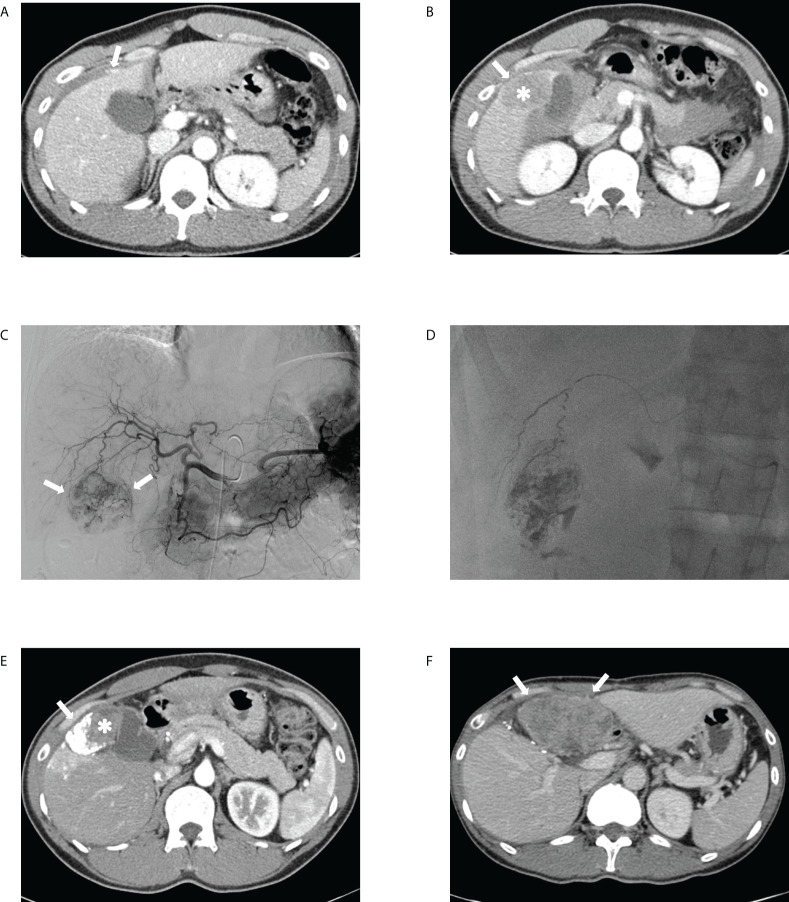
Transcatheter arterial chemoembolization (TACE) performed for ruptured hepatocellular carcinoma in a 41-year-old male patient. The portal phase of the initial computed tomography (CT) demonstrates **(A)** contrast media extravasation (arrow) from the tumor to the peritoneum and **(B)** washout lesion (asterisk) in segment 6 of the liver protruding to the peritoneum with focal discontinuity (arrow) of the liver surface, suggesting ruptured hepatocellular carcinoma. **(C)** Celiac angiography showing hypervascular tumor staining (arrows) corresponding to the CT findings. **(D)** Spot image of fluoroscopy demonstrating administration of iodized oil-doxorubicin mixture at the branch of S6 with partial uptake within the tumor. **(E)** Portal phase of the 1-month follow-up CT shows lipiodol-doxorubicin uptake (arrow) in the lateral aspect of the index tumor with a residual lesion (asterisk) in the medial portion. **(F)** Portal phase of the 11-month follow-up CT after tumorectomy performed at 6-month demonstrates a huge hypoattenuating mass (arrows) in the peritoneum, suggesting intraperitoneal drop metastasis.

Regarding the development of IPDM, 105 patients underwent follow-up CT or MR images after HCC rupture, and 16 patients (15.2%, 16/105) experienced new development of IPDM. The incidence of IPDM gradually increased and reached a plateau from 1 year after HCC rupture (**Figure 4A**). The 1-year. 2-year, and 5-year IPDM rates were 15.2%, 15.2%, and 18.2%, respectively. OS in patients without IPDM was significantly higher than that in patients with IPDM (median, 15.1 vs. 6.3 months) (**Figure 4B**). A representative case of IPDM is shown in **Figure 5**.

## Discussion

According to the results of this study, TAE/TACE provided effective immediate hemostasis with a low rebleeding rate and may serve as a bridge treatment before elective surgery. In addition, most cases of IPDM occur within 1 year; thus a more thorough surveillance should be considered for patients with spontaneous rupture of HCC, even after immediate hemostasis and eventual tumor control.

The characteristics of patients with ruptured HCC vary widely, and comparison of the treatment modalities in these patients most likely yield skewed results owing to the sampling bias. Commonly, patients with resectable tumors and preserved liver function undergo elective or staged surgery, whereas patients with unresectable tumors or poor liver function are more likely to undergo conservative treatment or TAE at best. Therefore, in our study, patients were divided into two groups: those who received treatment with palliative intent of immediate hemostasis and those who received treatment with therapeutic intent of hemostasis and ultimately tumor control. Laboratory results, tumor characteristics, and imaging findings were poorer in the palliative treatment group and those who received TAE/TACE than in the curative treatment group and those who underwent surgery (**Tables 1**, **2**).

Previous studies have reported rebleeding rate varying from as low as 1% to as high as 20% (12, 15). In our study, the overall rebleeding rate in patients who underwent TAE or TACE was 3.1%. The rate of rebleeding was not significantly different between the two groups based on treatment intent. In addition, 1-month survival rate of patients who underwent TAE or TACE was 94.8%, which is much higher than the previously reported rates, which ranged from 28.6% to 87.5% (16). With the advent of cone-beam CT, which increases the sensitivity of tumor and feeding artery detection (17), TAE/TACE may have a larger than previously anticipated role in effective hemostasis in the acute management of ruptured HCC.

The prognosis of ruptured HCC is poorer than that of non-ruptured HCC (2). In a systematic review of ruptured HCC, the overall aggregate 1-year survival was 46.4% (range, 17.5% to 90.1%) (16), which is comparable to our result of 41.3%. Based on the treatment modality, TAE/TACE and emergency or staged operations provided better survival than conservative treatment. While there was no statistical difference between OS in patients who underwent emergency surgery and that in those who underwent staged surgery (*p* < 0.606), patients who underwent TAE/TACE had poorer OS than those who underwent either emergency or staged surgery (*p* < 0.05). This result was concordant with those of the previous studies that reported a more favorable long-term outcome with surgical intervention (16). However, since TAE/TACE was the only treatment option for patients with unresectable tumors or poor liver function, a selection bias might have occurred in favor of patients with better liver function and tumor characteristics to undergo surgical management (**Table 2**). Comparing patients who underwent emergency operations and patients who underwent staged operations in terms of median survival, the latter group demonstrated a longer median overall survival (19.1 vs. 71.1 months, *p* = 0.606). All the baseline statistics of these two groups were not significantly different except for the tumor size (9.64 ± 3.64 and 6.72 ± 2.09 for the emergency operation group and the staged operation group, respectively) (*p* = 0.021). This may have contributed to longer overall survival for the staged operation group. In addition, the number of patients in each group were relatively small (14 for emergency operation and ten for staged operation). Thus, a more comprehensive study is needed to compare the staged operation and the emergency operation in the setting of ruptured HCC. Considering that TAE/TACE may be the only option for most patients because of either poor liver function or unfavorable tumor characteristics for surgical options and that TAE/TACE may act as a bridge to staged surgery after initial hemostasis, TAE/TACE should be considered as a viable treatment option.

According to a previous study on the clinical course of patients with IPDM, 14.3% of the cases occurred after spontaneous rupture of HCC (5). In another study that compared peritoneal metastasis after emergency or delayed hepatectomy for spontaneous rupture of HCC, 35.3% and 40.7% of patients from each group, respectively, developed IPDM (4). Sixteen of 105 patients (15.2%) developed IPDM during the follow-up period in our study, and 11 patients (69%) developed IPDM within 1 year. After cytoreductive surgery, the survival rate in patients with IPDM was better than that in patients with other extrahepatic recurrences (1- and 2-year survival rates of 83% and 71%, respectively) (18). Nonetheless, the presence of IPDM in patients with spontaneous rupture rendered significantly worse survival in our study (median survival: 15.1 vs. 6.3 months). Thus, close surveillance of patients with spontaneous rupture of HCC for IPDM needs to be considered for up to at least 1 year.

This study has several limitations. First, it was a retrospective study and the number of patients included was small, especially those who underwent emergent and staged operations. Hence, an effective subgroup analysis comparing TAE/TACE, emergency surgery, and staged surgery could not be performed. Second, the intent of the treatment was retrospectively determined because of the emergent nature of the ruptured HCC. Finally, the baseline demographics of patients who underwent interventional procedures and surgical operations were different, which hindered the accurate depiction and comparison of these two treatment modalities.

In conclusion, TAE/TACE provided immediate effective hemostasis with a low rebleeding rate and adequate 1-month survival rate. In addition, TAE/TACE may serve as a bridge to staged surgery in patients with resectable HCC and good liver function. After initial treatment, a closer surveillance should be considered for up to at least 1 year for a high probability of IPDM.

## Data availability statement

The raw data supporting the conclusions of this article will be made available by the authors, without undue reservation.

## Ethics statement

The studies involving human participants were reviewed and approved by Institutional Review Board of Seoul National University of Hospital. Written informed consent for participation was not required for this study in accordance with the national legislation and the institutional requirements.

## Author contributions

The authors confirm contribution to the paper as follows: Study conception and design: JP and JWCho; data collection: YSS and YSJ; analysis and interpretation of results: JP, JWCho, HCK, and JWChu; draft manuscript preparation: JP and JWCho. All authors reviewed the results and approved the final version of the manuscript.

## Conflict of interest

The authors declare that the research was conducted in the absence of any commercial or financial relationships that could be construed as a potential conflict of interest.

## Publisher’s note

All claims expressed in this article are solely those of the authors and do not necessarily represent those of their affiliated organizations, or those of the publisher, the editors and the reviewers. Any product that may be evaluated in this article, or claim that may be made by its manufacturer, is not guaranteed or endorsed by the publisher.

## Abbreviations

HCC, hepatocellular carcinoma; IPDM, intraperitoneal drop metastasis; OS, overall survival; TACE, transcatheter arterial chemoembolization; TAE, transcatheter arterial embolization.
