# Supplementary material for: Clinical course and role of embolization in patients with spontaneous rupture of hepatocellular carcinoma
Source: Front Oncol. 2022 Sep 5;12:999557. doi: 10.3389/fonc.2022.999557 (PMC9483098; doi:10.3389/fonc.2022.999557)
Supplement: Supplementary file 1 [file Table_1.docx]

**Supplementary table 1. Treatment modalities and outcomes**

|  | **Active treatment group (n=97)** | **Palliative treatment group (n=30)** | **P value** |
| --- | --- | --- | --- |
| **Treatment modalities** |  |  |  |
| **Conservative treatment** | 0 (0.0) | 17 (56.7) |  |
| **TAE or TACE** | 73 (75.3) | 13 (43.3) |  |
| **Emergency operation** | 14 (14.4) | 0 (0.0) |  |
| **Staged operation** | 10 (10.3) | 0 (0.0) |  |
| **One-month rebleeding rate** | 2 (2.1) | 1 (3.3) | 0.558 |
| **One-month mortality rate** | 3 (3.1) | 5 (16.7) | **0.018** |

TAE: transarterial embolization, TACE: transarterial chemoembolization
